# Differences in Social Determinants of Health between Urban Indigenous Migrants and Non-Indigenous People in North-Eastern Mexico: An Analysis to Prioritize

**DOI:** 10.3390/ijerph18168464

**Published:** 2021-08-11

**Authors:** Georgina Mayela Núñez-Rocha, Brenda Mayela Esqueda-Eguía, Ana María Salinas-Martínez, María Natividad Ávila-Ortiz, Ana Elisa Castro-Sánchez, Adriana Zambrano-Moreno, Karina Janett Hernández-Ruiz

**Affiliations:** 1 Facultad de Salud Pública y Nutrición, Universidad Autónoma de Nuevo León, Nuevo León 66455, Mexico; brenda.esquedaeg@uanl.edu.mx (B.M.E.-E.); amsalinasmartinez@gmail.com (A.M.S.-M.); natividad.avilao@uanl.mx (M.N.Á.-O.); ana.castrosh@uanl.edu.mx (A.E.C.-S.); adriana.zambranom@uanl.mx (A.Z.-M.); karina.hernandezr@uanl.mx (K.J.H.-R.); 2 Unidad de Investigación Epidemiológica y en Servicios de Salud, Instituto Mexicano del Seguro Social, Nuevo León 64360, Mexico

**Keywords:** social determinants of health, indigenous population, migration, vulnerable groups

## Abstract

The degree to which social determinants of health differ between indigenous migrants and non-indigenous people born and raised locally is currently unknown. We compared social determinants of health between indigenous migrants and non-indigenous people from urban north-eastern Mexico. Additionally, we ranked priorities for addressing the negative social determinants of health. This was a population-based comparative cross-sectional study (*n* = 235 indigenous migrants and 168 non-indigenous people). A two-stage non-random sampling was carried out from June to August of 2019. Heads of households ≥18 years and those with the ability to communicate in Spanish were recruited house by house. Structural and intermediary determinants of health were identified according to the World Health Organization Conceptual Framework and priorities were ranked using Z-scores. Being a migrant indigenous increased 1.6 times the odds of low education (95% CI = 1.1, 2.4). In addition, the migrant indigenous status increased the odds of poor housing, unhealthy behaviour and low social cohesion (*p* < 0.05). Housing, behaviours and health service accessibility were top priorities for indigenous migrants and structural determinants for non-indigenous people. The findings show that the right to access the social determinants of health has not yet been guaranteed for indigenous communities.

## 1. Introduction

The World Health Organization has called attention to social inequalities and the social determinants of health [1,2]. The social determinants of health are non-medical factors (education, income, job and food security, housing, non-discrimination and access to health services, among other) that influence health outcomes [3]. Social determinants of health may vary by indigenous status. Studies reveal that negative social determinants are greater in indigenous populations than in non-indigenous communities, even in countries with high human development [4,5]. A lack of social determinants of health creates undesirable circumstances such as disparities, discrimination and social injustice, all of which lead to poor health consequences over multiple generations. There are over 370 million indigenous peoples living in approximately 90 countries with low standards of health [6]. In Mexico, there are 68 indigenous groups and more than 25 million people who self-describe themselves as indigenous, a figure that represents 21.5% of the country’s total population. A high percentage do not have proper housing and have less access to education and formal jobs. Their health needs are high and their access to health care is poor, forcing them to migrate [7,8,9]. This country has documented 57.5% of indigenous people with poor access to basic housing and 31.5% with poor access to food compared to 15.5% and 29.2% of non-indigenous people, respectively. In addition, 40% of indigenous people live in extreme poverty and 31.1% are not formally educated compared to 14.3% and 15.4% of non-indigenous, respectively [10]. Inequality increases even more when indigenous people migrate in search of better opportunities. Migration mostly occurs from small rural towns to large, developed cities, which involves numerous risks for the social determinants of health. Indigenous migrants settle in marginalized areas, suffering discrimination, social undervaluation and labour exploitation; males are usually employed as construction workers or work in the informal economy, while females are mostly hired for domestic work, with no social benefits [8,11]. 

There is a gap in the knowledge about the social determinants of health in indigenous populations that migrate from rural to urban areas. Nuevo Leon is an example of such internal migration. This north-eastern state is highly industrialized and has become a destination for indigenous migrants [11]. They come from different parts of the country such as San Luis Potosí (*Tenek, Nahuas*), Querétaro (*Otomies*), Oaxaca (*Mixtecos, Triquis*) and Veracruz (*Nahuas*), among others. Here, there are 59,196 people who speak an indigenous language (1.2% of the state’s inhabitants) and its metropolitan area has reached an annual indigenous immigration growth rate of 12% [12]. However, it is not known to what degree the social determinants of health differ between indigenous migrants and non-indigenous people who are born and raised locally. Regions such as this require evidence for establishing public policies to correct inequalities before irreversible health outcomes occur. The objective of the present study was to compare social determinants of health between indigenous migrants and non-indigenous people from urban Nuevo Leon, Mexico. Additionally, we sought to rank the priorities for addressing the negative social determinants of health.

## 2. Materials and Methods

This was a population-based comparative cross-sectional study. A two-stage non-random sampling was carried out from June to August of 2019. The first phase consisted of identification and selection of geographical areas home to indigenous migrants and non-indigenous locals (target populations). Two adjacent neighbourhoods were located (one in front of the other); the indigenous migrant cluster was endorsed by a non-profit *Zihuame-Mochilla* organization and the non-indigenous cluster by local government authorities. The second phase consisted of recruiting, from house to house, the person that a given family recognized as the head of the household, i.e., the person who contributed the most to the family’s economy. Household heads were consecutively selected after having verified the selection criteria of minimum 18 years and being able to communicate in Spanish. If the head of the family was not available at the time of the visit, a second and third visit were scheduled. The response rate was 98% for indigenous migrants and 97% for non-indigenous people. Two eligible indigenous migrants’ household heads were excluded as they were under the influence of alcohol/psychotropic substances at the time of the interview. In a pilot study, we identified an average difference of 16 percentage points of six social determinants between indigenous migrants and non-indigenous people. We estimated 117 individuals as the minimum sample size in each group considering previous difference in the calculation with power of 80% and alpha of 0.05. We included 235 indigenous migrants and 168 non-indigenous. The protocol was approved by the Committee of Ethics and Health Research (17-FaSPyN-SA-13.TP). The corresponding informed consent was provided by all the participants and the Helsinki declaration was respected.

### 2.1. Indigenous Migrant and Non-Indigenous Status

There were three elements that defined an indigenous individual: (a) the use of an indigenous language, (b) self-ascription (that was, recognizing oneself as such) and (c) belonging to a home where there is a person who speaks an indigenous language. Migrant status was defined according to place of birth. The participants were categorized as indigenous migrants and non-indigenous (non-migrant).

### 2.2. Social Determinants of Health

Structural and intermediary determinants of health were distinguished as stated by the World Health Organization Conceptual Framework on the Social Determinants of Health [3]. Structural determinants consisted of education (absent, primary and secondary, or higher), occupation (informal or formal economy work), monthly family income (<2 or ≥2 minimum wages equivalent to 272.7 USD) and social protection (*Prospera* beneficiary or non-beneficiary). Four categories of intermediary determinants of health were included: (a) housing: ownership, metal-galvanized iron sheets in walls and roofs and overcrowding (≥3 persons-per-room; yes or no); (b) behavioural: daily tobacco smoking, excessive consumption of alcohol at least once a week, age ≤18 at first intercourse and contraceptive use at first intercourse (yes or no); (c) health service accessibility: affiliation with a public health care system, health care use in the last 12 months, has been ill ≥ 15 days without seeking health care, perceives long the traveling time between home and health care facility, perceives long the health care waiting time and work prevents from seeking health care (yes or no); (d) social cohesion: this was measured with the Neighbourhood Social Cohesion Scale Spanish version [13], which is based on the English version that has showed acceptable psychometric properties [14,15]. It consisted of four subscales: interpersonal trust (10 items, alpha = 0.75), sense of belonging (4 items, alpha = 0.55), participatory behaviour (7 items, alpha = 0.52) and shared identity (2 items, alpha = 0.75). Participants responded using a 5-point Likert scale. All items were written in the positive sense, with higher scores indicating greater social cohesion. Mean scores were transformed into a 0–10 scale and categorized as low (<4), moderate (4 to 7) and high (≥8).

### 2.3. Well-Being (Physical Health Status and Psychological Distress) 

History of previous diagnosis of diabetes mellitus, hypertension, heart disease and dyslipidaemia was identified, as was information on any disability (visual, hearing, or motor impairment). Psychological distress was measured with the Kessler K–10. This is a short screening scale with easy application that is used in population-based studies [16,17]. It has shown acceptable psychometric properties in Aboriginal Australians [18] and Mexicans (Spanish version) [19]. It consisted of 10 items related to anxiety and depression symptoms, with responses in a 5-point Likert scale (alpha = 0.83). All items were written in the positive sense, with higher scores indicating greater psychological distress. The minimum possible score was 10 and the maximum was 50. Answers were categorized into low (10–15), moderate (16–21), high (22–29) and very high psychological distress (30–50). 

### 2.4. Sociodemographic and Other Variables

Age, sex, marital status, years of education, type of family and head of the family (father or mother) were all considered as sociodemographic variables. Participants were interviewed by trained personnel (a medical doctor, a social worker and a nutritionist). Interviews ranged in duration from 15 to 20 min. At the end of an interview, height (in centimetres) and weight (in kilograms) were measured using a Taylor (^®^USA) portable digital scale (Oak Brook, Chicago, IL, USA), calibrated daily and a wall stadiometer. Measurements were taken without shoes and with light clothing, with feet together and heels, back and hips touching the wall. The body mass index was calculated as weight/height^2^ (kg/m^2^) and classified as follows: underweight or normal weight, <25 kg/m^2^; overweight, 25–29 kg/m^2^; obesity, ≥30 kg/m^2^.

### 2.5. Statistical Analysis

Frequencies were obtained for the categorical variables, as were means and standard deviations for the non-categorical variables. Differences between indigenous migrants and non-indigenous were analysed with the chi-square test and univariate odds ratios with 95% of confidence intervals (CI). Priorities were determined by social determinant of health and ranked according to Z-scores: Zi = (Xi − $\bar{X}$)/S, where Xi was the observed value of each measure, $\bar{X}$ was the average and S was the standard deviation. 

## 3. Results

The mother was the head of the family in 59.2% of indigenous migrant households and in 55.7% of non-indigenous homes; 42% of indigenous migrants were born in southeast Mexico and 88.2% spoke Nahuatl. Most of them had more than 20 years in the current address. There were no age differences between indigenous migrants and non-indigenous people (40.9 ± 14.2 vs. 42.4 ± 14.5 years, *p* > 0.05), but fewer indigenous migrants were married or had a nuclear family (45.9% vs. 58.3% and 58.3% vs. 76.2%, respectively; *p* < 0.05). They also had fewer years of education (7.2 ± 3.8 vs. 8.5 ± 3.4, *p* < 0.05).

### 3.1. Social Determinants of Health

All structural determinants were equivalent between indigenous migrants and non-indigenous people, except for one—being a migrant indigenous individual increased 1.6 times the odds of low education (Table 1). 

Most of the intermediary determinants were unfavourable for the indigenous migrant population; being an indigenous individual increased the odds of poor housing, unhealthy behaviour and low social cohesion (Table 2).

### 3.2. Well-Being (Physical Health and Psychological Distress)

History of health conditions was similar between indigenous migrants and non-indigenous people, except for diabetes. Being an indigenous migrant increased the odds of self-reported diabetes by up to 1.7 times. Up to one-third registered high or very high psychological distress and there were no differences by indigenous migrant and non-indigenous category (Table 3).

### 3.3. Priority Ranking

Housing, behaviours and health service accessibility were top priorities for indigenous migrants, as well as structural determinants for non-indigenous people (Table 4).

## 4. Discussion

This is the first study in Mexico to compare indigenous migrants and non-indigenous urban residents and rank priorities for addressing the negative social determinants of health. Education was the only structural determinant that differed in the indigenous migrant category; indigenous individuals had a 60% higher chance of receiving low education. Moreover, the frequency of low education was high in both populations, with values (55% and 44%) greater than the state-wide 25% and the nationwide 34% [20]. A study from southeast Mexico revealed that 85% of indigenous people and 75% of non-indigenous people had primary or no education [21]. For decades, low academic performance and high school dropout have been observed in indigenous communities. Migration from rural areas to cities makes it difficult to satisfy the educational demands of ethnically distinctive populations and intercultural education policies need to be reinforced [8,9]. Occupation and income were critical and more than 60% of our studied groups were in the informal economy and a high percentage of households had a low income, irrespective of indigenous or migrant status. People leave school to obtain a job to support the family economy; this is paradoxical, since lower education is strongly linked to poorly paid occupations. Mexico offers the Basic Education Scholarship Program and the Universal Scholarship Program for high school students who pursue equitable education, as well as for those who live in vulnerable conditions. Marketing strategies are important for allowing communities to take advantage of these resources, since the percentage of beneficiaries from the previous social program, called *Prospera*, was remarkably low. 

Various intermediate social determinants evidenced inequality. Housing was the highest priority addressed among indigenous migrants; an indigenous migrant was 5–6 times more likely to have a house made of poor-quality materials. Noticeably, neighbourhoods were close to each other, showing ancestral gaps had not yet been overcome. Housing remediation is not easy given financial constraints and interventions should consider public–private partnerships. Smoking, sex at young age and no contraceptive protection were also high priorities to be addressed among indigenous migrants. An indigenous migrant was twice as likely to be an active smoker. Prevalence of smoking was higher among indigenous migrants (45%) than at the state and national levels (25% and 17.6%, respectively) [22]. The south-eastern Mexican study showed that 15.9% and 9.4% of indigenous and non-indigenous people were smokers, respectively [21]. Nuevo Leon is one of the 11 states with smoke-free laws. Nationwide policies include restrictions on tobacco advertising, high purchase taxes and health warnings on packages. There is even a phone line to aid in stopping smoking [22]. Without a doubt, much remains to be achieved. Another behavioural social determinant was alcohol consumption. The difference in the frequency of drinkers between populations was marginal, though there was a tendency among indigenous migrants to be greater drinkers. Other studies have found no disparity between indigenous and non-indigenous groups [5,21]. Accessibility to health services was equivalent in all but one factor. A lack of utilization was high in both populations, but it was worse among non-indigenous people. Medical facilities being distant and not being able to miss work may have influenced these factors. The higher prevalence of diabetes among indigenous migrants may have led to more visits to the doctor. As such, the non-use rate was lower among this population; however, more research is needed to identify the source of these differences. Affiliation with a public health care system was remarkably high, irrespective of indigenous or migrant status; more than 8 of 10 were affiliated with a public health care system. The 2012 Health and Nutrition Survey showed that 73% of indigenous people and 77% of non-indigenous people were affiliated with a public health care system [23]. Mexico has implemented different government initiatives for highly marginalized groups to reduce vulnerability. The Instituto de Salud para el Bienestar was founded in 2020 to provide free health services to people who do not have social security medical services (IMSS or ISSSTE). 

Social cohesion is the bridge between structural and intermediary determinants. Three of four aspects of social cohesion differed between indigenous migrants and non-indigenous people. Markwick et al. [5] identified that 27.3% of Aboriginal Australians are untrusting compared to 20.9% of the non-Aboriginal population. In our study, frequencies were much higher: 65.2% of indigenous migrants and 49.4% of non-indigenous people had low interpersonal trust. Low trust may be a consequence of being a victim of discrimination and exploitation. Socially integrated communities share benefits. Social cohesion is important for the productive functioning of society and, therefore, health [24]. Regarding physical well-being, one-third of indigenous migrants and non-indigenous people had similar histories of hypertension, but self-reported diabetes was higher among indigenous migrants (37%), despite age and obesity similarities. The prevalence of diabetes among indigenous populations in Mexico ranges from 0 (Huicholes) to 26.2% (Mixtecos) [25]. The prevalence of diabetes among indigenous populations in Australia ranges from 3.5 to 33.1% [26]. In many developed countries, indigenous populations have higher prevalence of diabetes, but the pattern is inconsistent and differences may be attributed to adopted obesogenic lifestyles and genetic predispositions [26,27]. Jiménez et al. [21] identified a significant effect of the interaction between indigenous origin and family history on the risk of diabetes (odds ratio = 3.1, 95% CI 1.3–7.5). On the other hand, there is an increasing body of literature demonstrating that stressors that cause deterioration on the biological system may progress to negative long-term health outcomes. We identified one-third of indigenous migrants and non-indigenous people as having similar high or very high psychological distress. In Australia, being an indigenous person doubled this risk of psychological distress [5]. Interventions such as mindfulness-based stress reduction may be beneficial [28]. 

### Limitations of the Study

The study only included urban residents. Structural issues, health service accessibility and psychological distress, among other factors, may differ between urban and rural environments. Additionally, caution is needed when generalizing these results to rural residents. Self-reported data were subject to the obtaining of socially acceptable responses, e.g., behavioural questions; to obtain inaccurate responses to sensitive topics, e.g., family income question; or to obtain inaccurate responses due to memory recall or subjective perception, e.g., traveling and waiting times questions. Consequently, underreporting may be present. However, if bias did occur, it did so in both populations. Disparities cannot be attributed solely to ethnicity or migration; there are historical, political and sociocultural backgrounds that should also be considered. It is necessary to continue studying disparities between migrant populations.

## 5. Conclusions

Social determinants of health and their priority rankings differed between indigenous migrants and non-indigenous people from urban north-eastern Mexico. For indigenous migrants, disparities were evident in one structural social determinant (low education) and three intermediate (poor housing, non-healthy behaviours and poor access to health services) determinants. These findings show that the right to the social determinants of health has not yet been guaranteed for indigenous communities. Housing and behavioural factors are top priorities to be addressed among indigenous migrants. Public policies are required for reducing inequality gaps. The 2019–2024 Sectorial Health Program includes interculturality policies for fulfilling indigenous rights and guaranteeing comprehensive care [29]; these are major challenges for all levels of government. The question remains as to whether the measures will be able to finally close the historical gaps that have affected and continue to affect, indigenous peoples.

## Figures and Tables

**Table 1:** Structural determinants of health according to indigenous migrant and non-indigenous category.

Factor	Indigenous Migrants (*n* = 235) Number (%)	Non-Indigenous (*n* = 168) Number (%)	Odds Ratio	95% CI	*p* Value
None or primary education	129 (55.4)	73 (43.5)	1.6	1.1, 2.4	0.02
Informal economy worker	150 (64.4)	109 (64.9)	1.0	0.6, 1.5	0.92
Family monthly income <2 minimum wages ($272.7 USD)	111 (47.2)	67 (44.1)	1.5	0.9, 2.3	0.07
Non-beneficiary of the *Prospera* program	188 (80.7)	142 (84.5)	0.9	0.5, 1.3	0.32

**Table 2:** Intermediary determinants of health according to indigenous migrant and non-indigenous category.

Factor	Indigenous Migrants (*n* = 235) Number (%)	Non-Indigenous (*n* = 168) Number (%)	Odds Ratio	95% CI	*p* Value
**Housing**					
Not owned	103 (50.5)	51(36.7)	1.8	1.1, 2.7	0.01
Roof made of metal-galvanized iron sheets	89 (37.9)	16(9.5)	5.7	3.2, 10.3	0.0001
Walls made of metal-galvanized iron sheets	31 (13.2)	5 (3.0)	5.0	1.9, 13.0	0.0001
Overcrowding	41 (17.4)	10(6.0)	3.3	1.6, 6.9	0.001
**Behavioural**					
Smoking	105 (45.3)	52(31.0)	1.8	1.2,2.8	0.004
Drinking	135 (58.2)	81 (48.5)	1.5	0.9, 2.2	0.06
Age ≤ 18 at first intercourse	138 (61.9)	65 (41.9)	2.2	1.5, 3.4	0.001
No contraceptive use at first intercourse	184 (82.1)	110 (68.8)	2.1	1.3, 3.4	0.002
**Health service accessibility**					
Not affiliated with a public health care system	32 (13.7)	28 (16.7)	0.8	0.5, 1.4	0.42
Has not used health care in the last 12 months	141 (60.5)	118 (70.7)	0.6	0.4, 0.97	0.04
Has been ill ≥ 15 days without seeking health care	25 (27.5)	13 (26.5)	1.0	0.5, 2.3	0.90
Perceives long the traveling time between home and health care facility	49 (53.3)	32 (65.3)	0.6	0.3, 1.2	0.17
Perceives long the health care waiting time	44 (47.8)	29 (59.2)	0.6	0.3, 1.3	0.20
Work prevents from seeking health care	30 (37.0)	24 (42.1)	0.8	0.4, 1.6	0.55
**Social cohesion**					
Low interpersonal trust	152 (65.2)	83 (49.4)	1.9	1.3, 2.9	0.001
Low sense of belonging	108 (46.6)	48 (28.7)	2.2	1.4, 3.3	0.0001
Low participatory behaviour	213 (91.4)	160 (95.2)	0.5	0.2, 1.2	0.14
Low shared identity	179 (77.2)	112 (67.1)	1.7	1.1, 2.6	0.03

**Table 3:** Physical health and psychological distress according to indigenous migrant and non-indigenous category.

Indicator	Indigenous Migrants (*n* = 235) Number (%)	Non-Indigenous (*n* = 168) Number (%)	Odds Ratio	95% CI	*p* Value
Physical health					
Diabetes	86 (36.9)	42 (25.1)	1.7	1.1, 2.7	0.01
Hypertension	72 (30.9)	56 (33.5)	0.9	0.6, 1.4	0.58
Heart disease	17 (7.3)	9 (5.4)	1.4	0.6, 3.2	0.45
Dyslipidaemia	40 (17.2)	30 (18.0)	0.9	0.6, 1.6	0.84
Disability	23 (9.9)	14 (8.4)	1.2	0.6, 2.4	0.61
Overweight or obesity	183 (79.6)	130 (78.8)	1.0	0.6, 1.7	0.85
Psychological distress					
High or very high	72 (31.0)	41 (24.6)	1.4	0.9, 2.2	0.16

**Table 4:** Priority ranking for addressing social determinants and health needs according to indigenous migrant and non-indigenous category.

Factor	Indigenous Migrants’ Z Score ^a^	Priority Ranking	Non-Indigenous Z Score ^a^	Priority Ranking
Structural	0.0	4	0.0	1
Housing	−2.8	1	2.8	4
Behavioural	−2.8	1	1.4	2
Health services accessibility	−2.8	1	2.8	4
Social cohesion	−1.4	3	1.4	2
Physical health and/or psychological distress	−2.1	2	2.1	3

^a^ A high and negative Z score indicates higher priority.
